# 3D Model Characterization by 2D and 3D Imaging in t(14;18)-Positive B-NHL: Perspectives for In Vitro Drug Screens in Follicular Lymphoma

**DOI:** 10.3390/cancers13071490

**Published:** 2021-03-24

**Authors:** Fabien Gava, Carla Faria, Pauline Gravelle, Juan G. Valero, Cèlia Dobaño-López, Renaud Morin, Marine Norlund, Aurélie Gomes, Jean-Michel Lagarde, Cédric Rossi, Julie Bordenave, Laetitia Pieruccioni, Jacques Rouquette, Alba Matas-Céspedes, Jean-Jacques Fournié, Loïc Ysebaert, Camille Laurent, Patricia Pérez-Galán, Christine Bezombes

**Affiliations:** 1 Centre de Recherches en Cancérologie de Toulouse, INSERM UMR1037, CEDEX 1, 31037 Toulouse, France; fabien.gava@inserm.fr (F.G.); carla.faria@inserm.fr (C.F.); pauline.gravelle@inserm.fr (P.G.); julie.bordenave@inserm.fr (J.B.); jean-jacques.fournie@inserm.fr (J.-J.F.); ysebaert.loic@iuct-oncopole.fr (L.Y.); Laurent.Camille@iuct-oncopole.fr (C.L.); 2 Université Toulouse III Paul-Sabatier, CEDEX 9, 31062 Toulouse, France; 3 ERL 5294 CNRS, CEDEX 4, 31055 Toulouse, France; 4 Institut Universitaire du Cancer-Oncopole de Toulouse, CEDEX 9, 31059 Toulouse, France; 5 Laboratoire d’Excellence ‘TOUCAN-2’, CEDEX 1, 31037 Toulouse, France; 6 Institut Carnot Lymphome CALYM, 69495 Pierre-Bénite, France; 7 Department of Pathology, Institut Universitaire du Cancer de Toulouse, CEDEX 9, 31059 Toulouse, France; 8 Department of Hemato-Oncology, IDIBAPS, 08036 Barcelona, Spain; garcia32@clinic.cat (J.G.V.); cdobanol@clinic.cat (C.D.-L.); alba.matas87@gmail.com (A.M.-C.); PPEREZ@clinic.cat (P.P.-G.); 9 Centro de Investigación Biomédica en Red-Oncología (CIBERONC), 28029 Madrid, Spain; 10 IMACTIV-3D, 1 Place Pierre Potier, 31106 Toulouse, France; renaud.morin@imactiv-3d.com (R.M.); marine.norlund@imactiv-3d.com (M.N.); aurelie.gomes@imactiv-3d.com (A.G.); jean.michel.lagarde@imactiv-3d.com (J.-M.L.); 11 CHU Dijon, Hématologie clinique, Hôpital François Mitterand, 21000 Dijon, France; cedric.rossi@chu-dijon.fr; 12 RESTORE Research Center, Université de Toulouse, INSERM, CNRS, EFS, ENVT, 31100 Toulouse, France; Laetitia.PIERUCCIONI@cnrs.fr (L.P.); Jacques.ROUQUETTE@cnrs.fr (J.R.); 13 Department of Hematology, Institut Universitaire du Cancer de Toulouse, CEDEX 9, 31059 Toulouse, France

**Keywords:** follicular lymphoma, 3D model, spheroid, drug testing, 2D imaging, SPIM

## Abstract

**Simple Summary:**

Follicular lymphoma is an indolent B cell lymphoproliferative disorder of transformed follicular center B cells, which accounts for 20–30 percent of all non-Hodgkin lymphoma (NHL) cases. Although huge efforts have been made in the last 10 years, this pathology is still considered as incurable, leaving open the discovery and testing of new therapeutic targets requiring relevant preclinical models. Here, we report a realistic 3D model of t (14;18)-positive B-NHL cell culture (ultra-low attachment (ULA)-multicellular aggregates of lymphoma cells (MALC)), which monitored by state-of-the-art 2D and 3D imaging, allows more robust drug testing.

**Abstract:**

Follicular lymphoma (FL) is an indolent B cell lymphoproliferative disorder of transformed follicular center B cells, which accounts for 20–30 percent of all non-Hodgkin lymphoma (NHL) cases. Great advances have been made to identify the most relevant targets for precision therapy. However, no relevant models for in vitro studies have been developed or characterized in depth. To this purpose, we generated a 3D cell model from t(14;18)-positive B-NHL cell lines cultured in ultra-low attachment 96-well plates. Morphological features and cell growth behavior were evaluated by classical microscopy (2D imaging) and response to treatment with different drugs was evaluated by a high-content analysis system to determine the robustness of the model. We show that the ultra-low attachment (ULA) method allows the development of regular, spherical and viable ULA-multicellular aggregates of lymphoma cells (MALC). However, discrepancies in the results obtained after 2D imaging analyses on drug-treated ULA-MALC prompted us to develop 3D imaging and specific analyses. We show by using light sheet microscopy and specifically developed 3D imaging algorithms that 3D imaging and dedicated analyses are necessary to characterize morphological properties of 3D models and drug effects. This study proposes a new method, but also imaging tools and informatic solutions, developed for FL necessary for future preclinical studies.

## 1. Introduction

Follicular lymphoma (FL) is the second most common type of non-Hodgkin lymphoma (NHL). It is composed of malignant cells derived from germinal center B cells, both centrocytes and centroblasts, with a follicular growth pattern. In most cases, FL cells exhibit the t(14;18) translocation leading to the expression of the antiapoptotic Bcl-2 protein. It is located primarily in lymph nodes and characterized by a nodular pattern with variable-sized and usually closely packed follicles. FL usually has an indolent course and excellent overall survival. However, the disease remains incurable with conventional approaches and is characterized by repeated relapses [1,2]. Thus, FL research should focus on the development of new and more efficient molecules targeting key pathways leading to disease pathogenesis. To this end, relevant in vitro models should be amenable for testing new drugs in preclinical settings. Classically, these studies are performed with cancer cells cultured in suspension (2D), but 3D aggregate cultures, also named spheroids, are widely recognized as more physiologically relevant to normal and diseased human tissues [3]. Advantages for using spheroids in the study of solid cancers have been recognized for over 50 years [4] and their historical timeline was described recently [5,6,7]. These 3D models reproduce cell–cell and cell–matrix interactions, spatial organizations, mechanical constraints, nutrients and O_2_ gradients, critical parameters able to influence the biology of the disease and the response to treatments. Spheroids are essential for in vitro studies, filling the gap between conventional 2D cultures, from which they are very different [8], and animal models. They also offer useful properties for drug screening [9,10,11,12,13]. Although these 3D cell cultures are routinely used for the study of solid cancers, in NHL and more particularly FL, very few models exist. We are pioneers in the development of MALC (multicellular aggregates of lymphoma cells) models with the hanging drop (HD) method in 24-well plates (HD-MALC) with t(14;18)-positive B-NHL cell lines. This 3D model exhibits transcriptomic profiles similar to FL patients with an overexpression of gene families involved in survival pathways, including the NF-κB pathway, cell cycle regulation or hypoxic responses [14,15,16]. These models can be easily cocultured with cytotoxic immune cells such as NK or T lymphocytes, allowing the study of anti-CD20 antibody responses, drug penetration, immune cell infiltration and immune-escape mechanisms targetable by immunotherapy [17,18]. Although the HD method allowed a better understanding of FL biology and drug responses in a more relevant model than 2D cultures, it is not suitable for drug screening due to the manual transfer of neoaggregates into agarose-precoated wells. Moreover, one could speculate that methylcellulose (MC), which is added for HD-MALC formation, may induce matrix-driven alterations in growth, expression profiles, cell behavior or in drug responses. This has never been explored.

In order to avoid these biases and to improve the throughput necessary for testing drug efficacy, we adapted a MC-free method of t(14;18)-positive B-NHL cell culture in ultra-low attachment (ULA) 96-well plates where cells were induced to self-aggregate into MALC (ULA-MALC). Morphological features and cell growth behavior were evaluated by classical microscopy (2D imaging) and response to treatment with different drugs was evaluated by a high-content analysis system to determine the robustness of the model. Furthermore, 3D imaging and 3D analyses were specifically developed to characterize in depth the ULA-MALC model and drug effects.

## 2. Materials and Methods

### 2.1. Cell Lines and Drugs

The human aggressive t(14;18)-positive B-NHL cell line RL (t14:18; CD19+; CD20+; CD21+; CD22+; Hle-1+; HLA DQ+; HLA DR+; CD25-) was purchased from the American Type Culture Collection (ATCC, Rockville, MD, USA) and the transformed FL cell lines DOHH2 (t14:18; CD3^−^; CD10^+^; CD13^−^, CD19^+^; CD20^+^; CD34^−^, CD37^+^; CD38^+^; CD80^+^; CD138 ^−^, HLA-DR^+^), WSU-NHL (t14:18; CD3^−^; CD10^+^; CD13^−^; CD19^+^; CD20^+^; CD34^−^; CD37^+^; CD38^+^; CD80^+^; HLA-DR^+^) and SC-1 (CD3^−^; CD10^+^; CD13^−^; CD19+; CD34^−^; CD37^+^; CD38^+^; cyCD79a(^+^); CD80^+^; CD138^+^; HLA-DR^+^) were obtained from the DSMZ Collection (Braunschweig, Germany). These cell lines carry additional alterations besides BCL-2 overexpression due to t(14;18): RL carries p53/mut [19], whereas DOHH2 and SC-1 show Myc amplification DSMZ refs ACC47 and ACC558 respectively).

The cells were cultured in suspension (referred to as 2D culture) in complete RPMI 1640 medium supplemented with glutamine (Dominique Dutscher, Brumath, France) with 10% of FBS (Life Technologies, Villebon sur Yvette, France) and 1% of penicillin/streptomycin (Sigma-Aldrich, St Quentin Fallavier, France) at 37 °C in a humidified atmosphere containing 5% CO_2_. Mycoplasma contamination was routinely tested by using Mycoplasma Alert (Lonza, Basel, Switzerland).

Rituximab (RTX, Mabthera) and obinutuzumab (GA101) were provided by Roche (Boulogne-Billancourt, France) and Roche (Zurich, Switzerland) respectively. Venetoclax (ABT-199, Bcl2 inhibitor), rapamycin (mTOR inhibitor), ibrutinib (BTK inhibitor), lenalidomide (immunomodulator) and bendamustin (alkylating agent) were all purchased from Euromedex (Souffelweyersheim, France).

### 2.2. ULA-MALC Generation

Of the complete medium 100 µL containing 2500, 5000 or 10,000 FL cells were seeded in 96-well round bottom ULA plates (Corning, Samois sur Seine, France), centrifuged and cultured at 37 °C in a humidified 5% CO_2_ atmosphere. Of fresh medium 100 µL was added every 3 days to avoid spheroid shrinking and to counterbalance liquid evaporation.

### 2.3. ULA-MALC Characterization by 2D Imaging

After 3, 6 or 9 days of culture, ULA-MALC was visualized by bright field (BF) and fluorescent microscopy on an automated spinning disk confocal HCS device equipped with a 5× objective (Operetta, Perkin Elmer, Villebon sur Yvette, France). To visualize cell death, propidium iodide (PI) at 1 µg/mL (Life technologies, Villebon sur Yvette, France) was added directly into the wells for 1 h prior to imaging. For each well, 1 field and 16 stacks per field (10 µm step) were acquired. Morphological parameters (BF area) and cell death (PI area and PI intensity and laser 532 nm) were determined on stacks of images combined with maximum projection and analyzed by the Columbus software. Cell death was measured in relation to the total BF area.

Two-dimensional imaging was also performed using an inverted microscope (Nikon Eclipse TE200) and several morphological parameters were extracted based on an adapted macro from [20,21,22]:

- Projected area was calculated as previously described [21,22] according to the formula:
  (1) $$R=\sqrt{S/\pi }$$
  R = radius and S = measured area of 2D projection).
- Volume was calculated as described [23] according to the formula:
  (2) $$V=\frac{4}{3}\pi R^{3}$$
- Sphericity index (SI): the spherical geometry shape was calculated according to Equations (3) and (4):
  (3) $$\mathrm{Cir}=\frac{4\pi \cdot \mathrm{Area}}{{\mathrm{Perimeter}}^{2}}$$
  (4) $$\mathrm{SI}=\sqrt{\mathrm{Cir}}$$
  ULA-MALC were considered spherical when SI = 1.
- Roundness index (RI) corresponded to the circularity of the ULA-MALC where a perfect circle = 1.
- Solidity is an indicator of the roughness of the spheroidal surface. This index was determined to assess its regularity.

For live imaging, ULA-MALC from centrifugation to day 1 of culture were observed with BF (4× magnification) with Incucyte S3 Live-Cell Analysis System (Sartorius, Göttingen, Germany) or Cytation TM 1 (Biotek, Winooski, VT, USA) at 37 °C and 5% CO_2_.

### 2.4. ULA-MALC Characterization by 3D Imaging

ULA-MALC were fixed at different days of culture directly in the wells with 4% PFA (Alfa Aesar, Haverhill, MA, USA) overnight at 4 °C and rinsed with PBS. Nuclei were labeled with 1 µg/mL propidium iodide for 1 h at room temperature. ULA-MALC were then rinsed with PBS and included in 1% low-melting agarose (Life Technologies). 8 mm disks were punched and cleared with the methanol-benzyl alcohol/benzyl benzoate (BABB) technique as previously described [24,25]. Cleared samples were immersed in a quartz chamber filled with BABB during acquisition due to a 3D-printed disk holder. Acquisitions were performed with selective plane illumination microscope (SPIM) technology [26]. Two microscopes were used: SPIM [27] for MALC from day 1 to day 4 and MacroSPIM [28,29] only for MALC at day 5 and day 6. An ULA-MALC at day 2 of culture was used to check for potential differences between image acquisitions by SPIM and MacroSPIM. For both microscopes, 2 µm-step Z-stacks at 5× magnification with a pixel size of 1.3 µm for SPIM and 1.28 µm for MacroSPIM were generated. Driving module/software for those microscopes have been developed by INSCOPER (Rennes, France). Open-source image processing package ImageJ and IMARIS 7 software (BitPlane, South Windsor, CT, USA) were used.

An image processing pipeline was specifically developed and implemented to automatically segment the ULA-MALC samples in 3D from SPIM and MacroSPIM acquisitions. The images were first resampled to obtain a 3D isotropic resolution sufficient for morphological characterization. A 3D non-local denoising algorithm was then used to improve the signal-to-noise ratio, with the degree of smoothing automatically estimated with respect to the standard deviation of noise in the image. A segmentation based on hysteresis thresholding was finally implemented, performing a dual thresholding to reduce isolated pixels and improve the connectivity of the resulting segmentation. This method is particularly suited for the segmentation of ULA-MALC MacroSPIM images since the distribution of the gray levels in such images can be bivariate (background and homogeneous sample) or trivariate (background and heterogeneous sample with high and low intensity regions). The lower and upper thresholds used in hysteresis segmentation were initialized using values centered around the threshold estimated by the classical Otsu method [30], which minimizes the intraclass variance of the resulting segmentation. The algorithm’s output is a 3D binary mask image from which various 3D morphological parameters were estimated:

- Volume.
- Eccentricity:
  (5) $$e1=\sqrt{1-\frac{c^{2}}{a^{2}}}$$
  (6) $$e2=\sqrt{1-\frac{c^{2}}{b^{2}}}$$
  with *a*: longest ellipsoid axis, *b*: second longest ellipsoid axis, *c*: shortest ellipsoid axis. Eccentricity = 0 for a perfect sphere and increases following ellipsoid deformation with a maximum of 1.
- Sphericity:
  (7) $$S=36\pi \frac{{\mathrm{Volume}}^{2}}{{\mathrm{Surface}}^{3}}$$
- A sphere is considered perfect when S = 1 and decreases with the rugosity or deformation of the shape.
- Roundness:
  (8) $$R=\frac{6}{\pi }\cdot \frac{\mathrm{Volume}}{{\mathrm{majoraxis}}^{3}}$$
- Roundness = 1 for a perfect sphere and decreases with deformation.

### 2.5. Visualization of Proliferative Cells in Whole ULA-MALC

To visualize proliferative cells in whole ULA-MALC, we adapted protocols from [20,31] as follows: after 3 or 6 days of culture, ULA-MALC (2500 cells seeding) were fixed as described in the previous section. ULA-MALC were then incubated in a blocking solution composed of PBS/FBS 1%/Triton X-100 0.3% for 8 h at room temperature (RT) under mild agitation on an orbital shaker. Incubation with the primary antibody (mouse anti-human Ki67 clone MIB-1, Agilent technologies, Les Ulis, France), diluted at 1/100e in blocking solution, was performed for 3 days at RT under mild agitation. After several washes with PBS during 24 h, the secondary antibody (goat anti-mouse Alexa Fluor 488 IgG, Life Technologies), diluted at 1/100e in blocking solution, was incubated for 3 days at RT under mild agitation. After 24 h of washes in PBS, Ki67+ cells reflecting proliferative cells were visualized by 2D (Operetta) or 3D imaging (SPIM) as described in the previous sections.

### 2.6. Determination of Cell Death by Flow Cytometry

After 3 or 6 days of treatment, 3 ULA-MALC were pooled, mechanically dissociated, washed in PBS and transferred into FACs analysis tubes. Of 7-aminoactinomycin D (7AAD, BD Biosciences, Le Pont de Claix, France) 5 µL was then added according to the manufacturer’s instructions and dissociated cells were analyzed on a LSRII flow cytometer (BD Biosciences). Dead cells (7AAD^+^) versus living cells (7AAD) were analyzed by Cytobank.

### 2.7. Determination of Cell Viability by Trypan Blue Assay

After 3 or 6 days of culture, five pooled ULA-MALC were mechanically dissociated and cells were counted by trypan blue assay on a Malassez cell. The cell viability was determined with the formula: % of viability = (number of live cells/(number of live cells + number of dead cells)) × 100 where dead cells incorporate trypan blue.

### 2.8. Statistics

**Figure 1:**
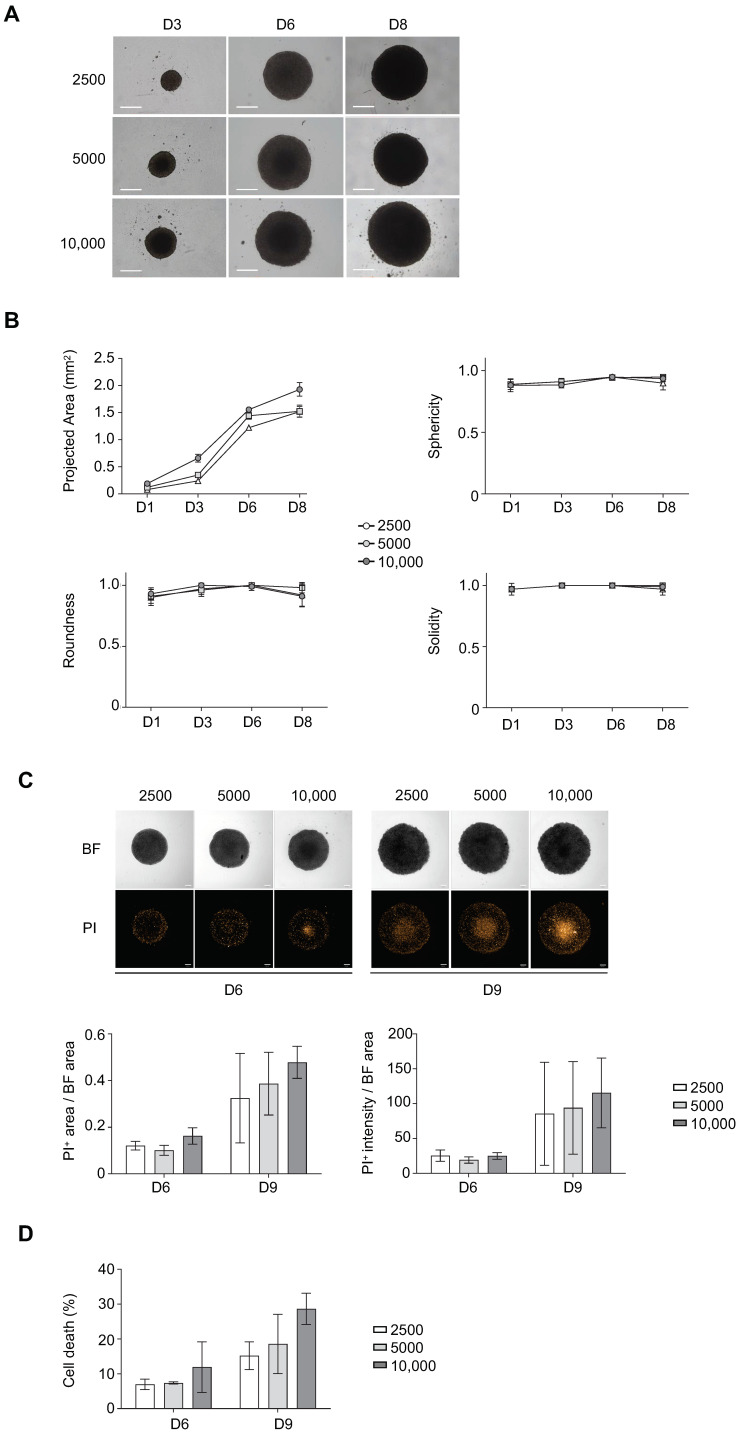
Influence of cell density on ultra-low attachment (ULA)-multicellular aggregates of lymphoma cells (MALC) biology. ULA-MALC established with RL cells was cultured at different cell seeding densities (2500; 5000 and 10,000 cells) and different biological characteristics were determined at different culture times. (**A**) Growth and morphology observed by bright field (BF) microscopy at 4× after 3, 6 and 8 days (D) of culture. Scale: 500 µm. These pictures are representative of 3 independent experiments each comprising 6 individual ULA-MALC. (**B**) Morphological properties (projected area, sphericity, roundness and solidity) determined after 1, 3, 6 and 8 days (D) of culture by 2D imaging analysis with the specific macro developed (see Material and Methods section). These graphs are the mean ± SD of *n* = 3 independent experiments each comprising at least *n* = 10 individual ULA-MALC. (**C**) Cell death visualization and quantification on whole ULA-MALC at day 6 and 9 following propidium iodide (PI) labeling and 2D imaging. Experiment performed on 3 independent experiments each comprising 7 individual ULA-MALC. Upper panel, representative pictures of bright field (BF) or propidium iodide (PI) at 5× magnification, scale: 200 µm. Lower panel, mean ± SD of the ratio of area or intensity of propidium iodide (PI) in relation to the bright field (BF) area. (**D**) Cell death quantification by flow cytometry after 7AAD labeling of dissociated MALC. This graph represents mean ± SD of the percentage of cell death (7AAD+) measured in 3 independent experiments of 3 pooled ULA-MALC.

**Figure 2:**
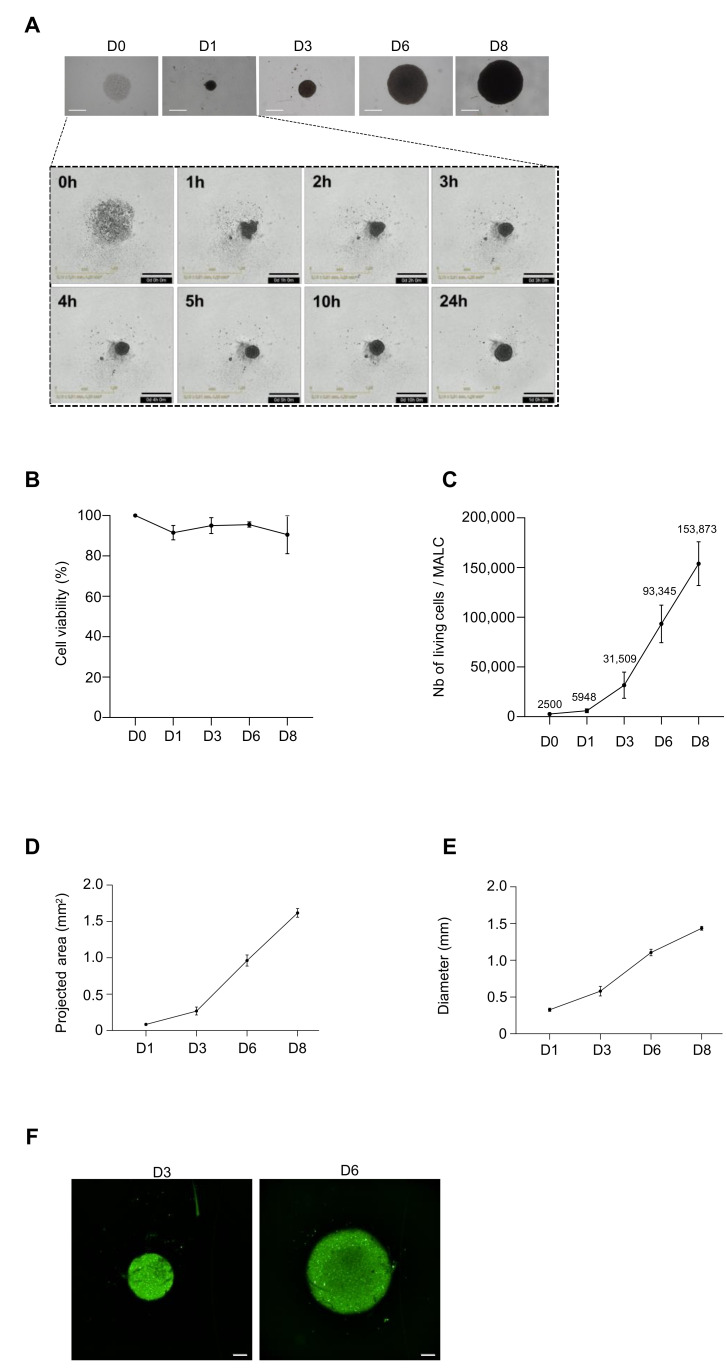
Two-dimensional characterization of ULA-MALC at 2500 cell seeding density. ULA-MALC established with RL cells were cultured at 2500 cell seeding density and observed at different times (D = day) by 2D imaging. (**A**) Upper panel, bright field (BF) pictures of ULA-MALC observed from day 0 to day 8 with an inverted microscope. Magnification 4×, scale 500 µm. Lower panel, ULA-MALC aggregation observed during the first 24 h at the indicated times (h = hour). Magnification: 4×, scale: 500 µm. Pictures are representative of 3 independent experiments each comprising 30 individual ULA-MALC. (**B**) ULA-MALC viability determined by Trypan blue exclusion assay. Results are expressed in percentage of viable cells in relation to day 0 (D0) and represent the mean ± SD of 3 independent experiments each comprising at least 11 individual ULA-MALC. (**C**) Living cells in ULA-MALC determined by Trypan blue exclusion assay. Results are expressed in number (Nb) of living cells per ULA-MALC and represent the mean ± SD of 3 independent experiments each comprising at least 11 individual ULA-MALC. Exact number of live cells is presented in the figure at each culture time. (**D**,**E**) Projected area (D) and diameter (E) of ULA-MALC measured at day 1, 3, 6 and 8 of culture by 2D imaging at 4× magnification. These graphs represent the mean ± SD of 3 independent experiments each comprising at least 30 individual ULA-MALC. (**F**) Ki67 labeling visualized 2D imaging (magnification 5×, scale: 200 µm) on whole ULA-MALC at day 3 and 6 of culture. Pictures are representative of 2 independent experiments each comprising 3 individual ULA-MALC.

**Figure 3:**
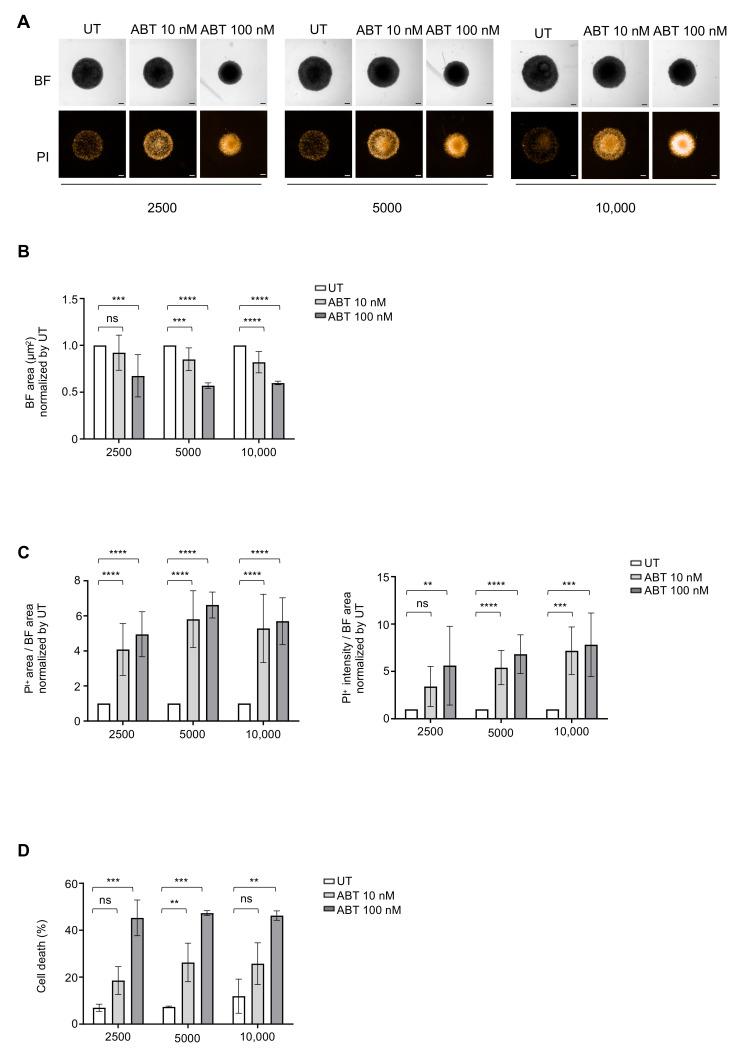
Influence of cell density on drug response after 3 days of treatment. ULA-MALC were seeded at different cell densities (2500; 5000 and 10,000 cells) and treated or not (UT) at day 3 of culture with ABT-199 (ABT) at 10 nM and 100 nM. Cell death was visualized and measured 3 days post-treatment (i.e., D6 of culture). Results are representative (pictures) or the mean ± SD (graphs) of 3 independent experiments each comprising 5 individual ULA-MALC. *p* values: **** = *p* < 0.0001, *** = *p* < 0.0005 and ** = *p* < 0.01 (**A**) Global morphology (BF) and propidium iodide (PI) labeling were visualized by 2D imaging, 5× magnification and scale: 200 µm. (**B**) Bright field (BF) area measured after 3 days of treatment. Results represent the mean ± SD of the global BF area normalized to the untreated condition (UT). (**C**) Quantification of cell death after propidium iodide (PI) labeling on whole ULA-MALC cultured at 2500, 5000 and 10,000 cell seeding densities and treated or not (UT) with ABT-199. PI area in relation to BF area (left panel) and PI intensity in relation to BF area (right panel), all normalized to the untreated condition (UT). (**D**) Cell death measured by flow cytometry in dissociated ULA-MALC cultured at 2500, 5000 and 10,000 cell seeding densities and treated or not (UT) with ABT-199. Results represent the mean ± SD of the percentage of cell death (7AAD^+^).

**Figure 4:**
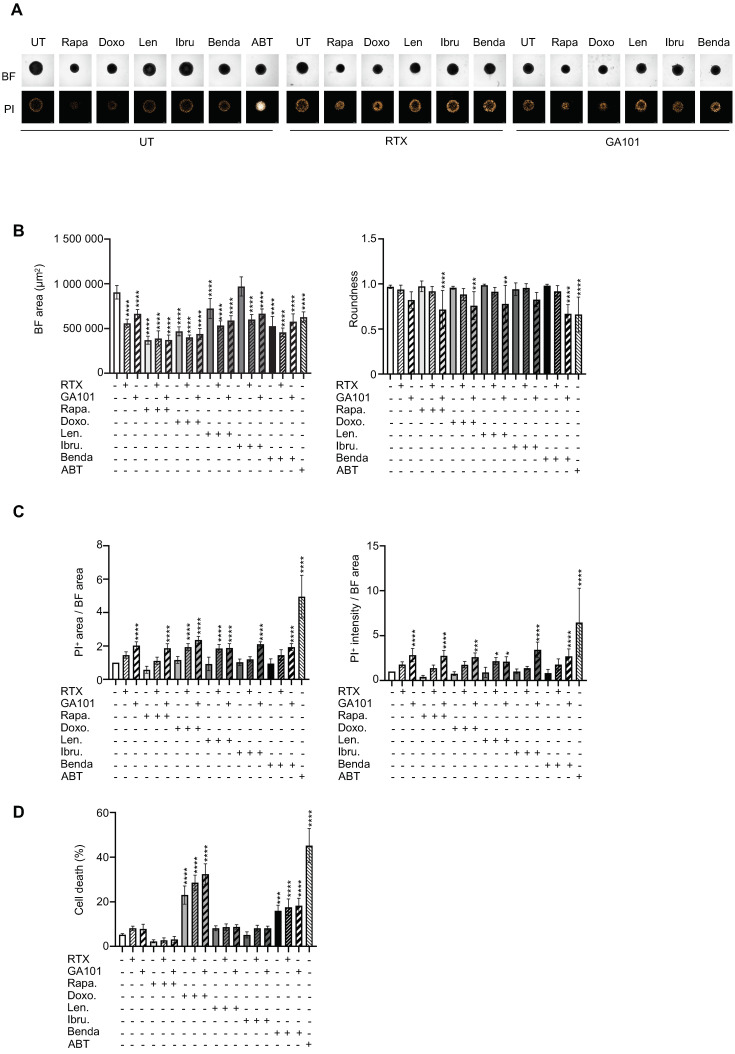
Drug response of ULA-MALC after 3 days of treatment. ULA-MALC were seeded at 2500 cells and treated or not (UT) after 3 days of culture with rapamycin (Rapa, 10 nM), doxorubicin (Doxo, 0.1 µM), lenalidomide (Len, 5 µM), ibrutinib (Ibru, 500 nM), bendamustine (Benda, 10µg/mL) in combination or not with rituximab (RTX) or GA101 (10 µg/mL). ABT-199 (ABT) at 100 nM was used as the positive control. Figures represent results obtained after 3 days of treatment and are representative (pictures) or the mean ± SD (graphs) of 3 independent experiments each comprising 5 individual ULA-MALC. *p* values: **** = *p* < 0.0001, *** = *p* < 0.0005, ** = *p* < 0.01 and * = *p* < 0.05. (**A**) Visualization of global morphology (BF) and propidium iodide (PI) labeling by 2D imaging, 5× magnification and scale: 200 µm. (**B**) Bright field (BF) area and roundness determined by 2D imaging analysis with the specific macro developed (see the Methods section). Results represent the mean ± SD. (**C**) Cell death quantification after PI labeling by 2D imaging on whole ULA-MALC. Results represent the mean ± SD of PI area in relation to BF area (left) and PI intensity in relation to BF area (right), all normalized to the untreated condition. (**D**) Cell death quantification by flow cytometry in dissociated ULA-MALC. Results represent the mean ± SD of the percentage of cell death (7AAD^+^).

**Figure 5:**
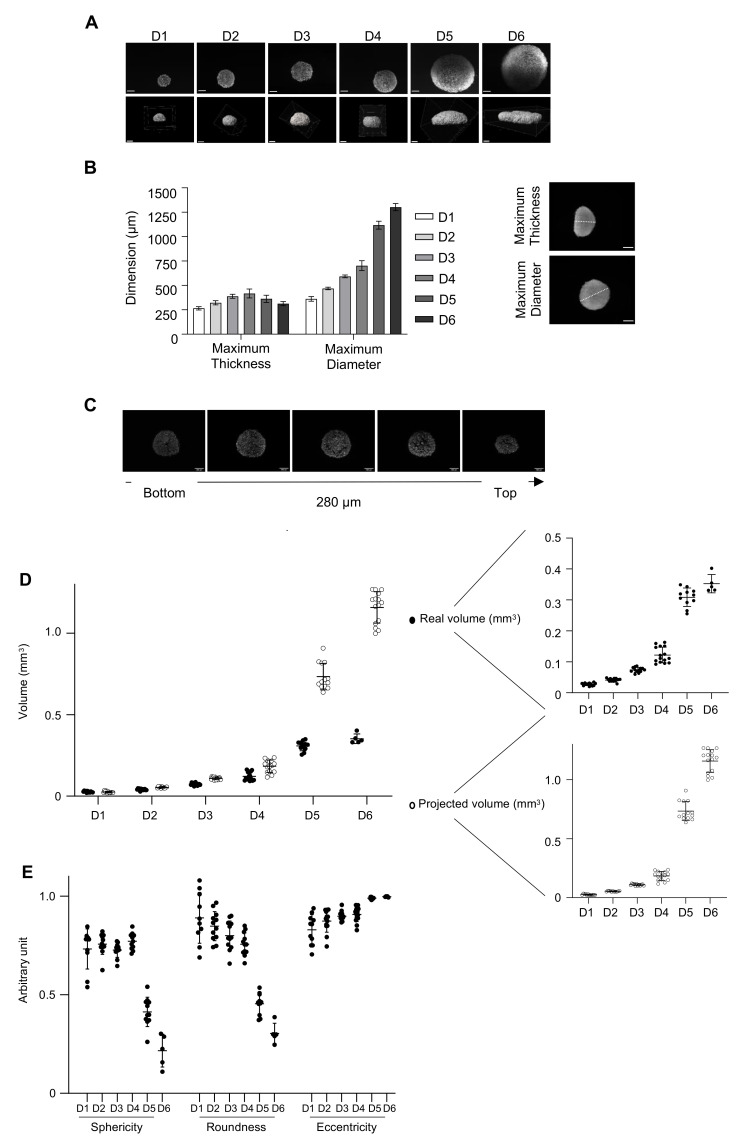
Three-dimensional characterization of ULA-MALC. ULA-MALC were cultured at a 2500 cell seeding density and observed at different times (D = day) by selective plane illumination microscope (SPIM) microscopy. (**A**) Pictures of center (upper panel) and 3D representation (lower panel) of ULA-MALC cultured during the indicated times (5× magnification, scale: 200 µm). These pictures are representative of 12–15 individual ULA-MALC (depending on the condition). (**B**) Quantification of maximum thickness and maximum diameter of ULA-MALC cultured during the indicated times. Histograms represent the mean ± SD of 12–15 individual ULA-MALC (depending on the condition). Insert, representative images of ULA-MALC at day 3 of culture (5× magnification, scale 200 µm). The white line represents the maximum thickness (upper) and maximum diameter (lower) used for quantification. (**C**) Ki67 labeling visualization by 3D imaging in ULA-MALC at day 3 of culture. Images were extracted from SPIM z-stack, from the bottom (left) to the top (right) of the MALC with a difference of 70 µm between them. Magnification 5×, scale: 200 µm. These pictures are representative of 2 independent experiments comprising 3 individual ULA-MALC. (**D**) Projected and real volumes of ULA-MALC after different days (D) of culture. Results are presented on global (left) or individual graphs (right) and are expressed by the mean ±SD of 5–15 individual ULA-MALC (depending on the condition). (**E**) Sphericity, roundness and eccentricity measured on ULA-MALC cultured during the indicated times (D = day). The graph represents the mean of each parameter ± SD of 5–15 individual ULA-MALC (depending on the condition).

**Figure 6:**
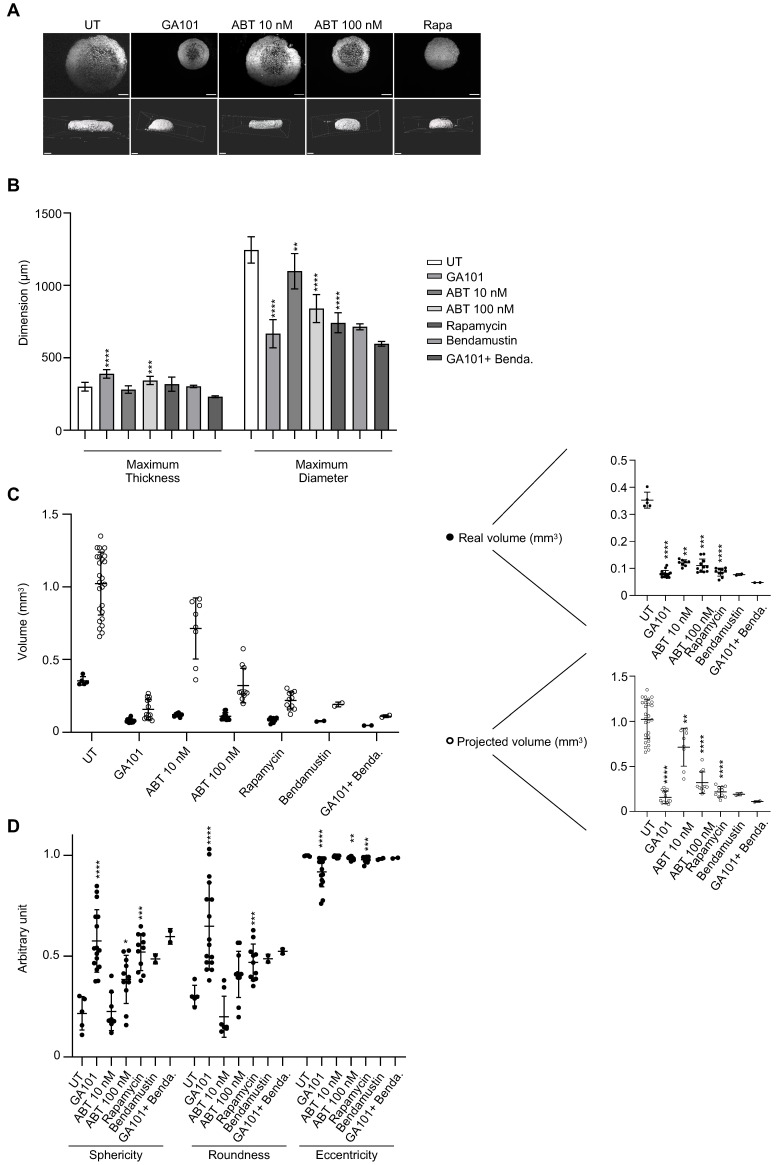
Three-dimensional characterization of drug effect. ULA-MALC (2500 cell seeding density) at day 3 of culture were treated or not (UT) with GA101 (10 µg/mL), ABT-199 (ABT, 10 or 100 nM), rapamycin (Rapa 50 nM), bendamustine (Benda 10 nM) and observed after 3 days by SPIM microscopy. *p* values: **** = *p* < 0.0001, *** = *p* < 0.0005, ** = *p* < 0.01 and * = *p* < 0.05. Bendamustin and GA101 + Bendamustin conditions were excluded from statistical tests due to a low number of replicates (*n* = 2). (**A**) Pictures of center (upper) and 3D representation (lower) of ULA-MALC treated or not (UT) with the indicated drugs (5× magnification, scale: 200 µm). These pictures are representative of 5–6 independent experiments comprising 8–25 individual ULA-MALC (depending on the condition). (**B**) Quantification of maximum thickness and maximum diameter of treated or not (UT) ULA-MALC. Histograms are the mean ± SD of 1–6 independent experiments comprising 2–25 individual ULA-MALC (depending on the condition). (**C**) Projected and real volumes of ULA-MALC treated or not (UT) with different drugs. Results are presented on global (left) or individual graphs (right) and are the mean ± SD of 1–6 independent experiments comprising 2–25 individual ULA-MALC (depending on the condition). (**D**) Sphericity, roundness and eccentricity of ULA-MALC treated or not (UT) with different drugs. The graph represents the mean of each parameter ± SD of 1–6 independent experiments comprising 2–11 individual ULA-MALC (depending on the condition).

For all the results obtained and presented in Figures 1–6 and Figures S1–S4, we applied various statistical analysis. Data shown Figures 1 and 2 and Figure S1 are means ± SD. For comparing three or more parameters, one-way ANOVA was used in Figures 3 and 4, Figures S3 and S4 whilst side-by-side Mann–Whitney tests were used in Figure 6. All tests were performed with GraphPad Prism software. *p* values: **** = *p* < 0.0001, *** = *p* < 0.0005, ** = *p* < 0.01 and * = *p* < 0.05.

## 3. Results

### 3.1. Determination of Optimal Cell Seeding Density for ULA-MALC Formation

Cell seeding density to perform 3D cultures can influence cell behavior and drug response [20,22], we therefore tested several conditions for optimizing ULA-MALC formation. ULA-MALC were first established with the RL t(14;18)-positive B-NHL cell line at 2500, 5000 or 10,000 cells/well and were morphologically observed during several days of culture (Figure 1). For all cell seeding densities, rapid and constant growth of single ULA-MALC per well was observed with a compact and round-type morphology that was apparently stable from day 3 to day 8 (Figure 1A). Parameters classically used for spheroid characterization such as sphericity, roundness, solidity and area (assuming that ULA-MALC were perfect spheres, see Material and Methods) were analyzed after bright field imaging and determined according to [22] (Figure 1B). The area varied between 0.08 and 1.5 mm^2^ from day 1 to day 8 of culture for 2500, 0.12 and 1.52 mm^2^ for 5000 and 0.19 and 1.9 mm^2^ for 10,000 cell densities. For all densities, ULA-MALC appeared spherical (SI > 0.9) and circular (RI > 0.9) with a regular surface (solidity index > 0.9) during cell culture. These culture conditions ensured the low variability required for in vitro assays as previously commented [10,32,33]. Cell death was determined on whole ULA-MALC by 2D imaging (Figure 1C) or on dissociated ULA-MALC using flow cytometry (Figure 1D). After 6 days of culture, ULA-MALC developed with 2500 or 5000 seeding concentrations exhibited a very low level of cell death as attested by the determination of PI area and intensity and 7AAD^+^ cells (less than 10%). However, with 10,000 cells, we observed an increase in basal cell death and result variability as attested by the higher standard deviation. After 9 days of culture, cell death increased in a concentration dependent manner with a higher variability.

Altogether, 2500–5000 cells would be the optimal cell seeding to modelize a well formed RL-ULA-MALC up to 6 days of culture. Similar results were obtained with three other t(14;18)-positive B-NHL cell lines (DOHH2, WSU-NHL and SC-1) (Figure S1). However, ULA-MALC performed with RL were more cohesive, thus prompting us to pursue our investigation with this cell line.

### 3.2. Biological Characterization of ULA-MALC

Live imaging performed in the first hours of culture showed that RL cells (2500 cell seeding density) aggregated spontaneously after the centrifugation step (day 0) (Figure 2A, insert) and then entered into a spheroidization process [13] consisting of aggregate compaction with an observable decrease of ULA-MALC size during the first days of culture followed by a constant growth (Figure 2A). For longer culture times, round-type morphology was still observed with a ULA-MALC diameter evolving between 320 µm at day 1, 500 µm at day 3, 1200 µm at day 6 and 1320 µm at day 8 (Figure 2A). Cell viability oscillated between 90 and 100% during the first 6 days of culture and then decreased after 8 days of culture (Figure 2B). The number of living cells per ULA-MALC increased from roughly 6000 cells at day 1 to approximately 32,000 cells at day 3, exponentially reaching 150,000 cells/ ULA-MALC after 8 days of culture (Figure 2C). At this cell density, the MALC area exponentially grew from 0.085 mm^2^ at day 1 to 1.6 mm^2^ at day 8 of culture (Figure 2D), concomitantly with its diameter (Figure 2E). Visualization of proliferative cells in whole ULA-MALC was determined by Ki67 detection after 3 or 6 days of 3D culture (Figure 2F). Ki67 labeling was homogeneously distributed thus showing that, in contrast to solid cancers [34,35,36,37], no regionalization of proliferation appeared in the t(14;18)-positive B-NHL cell 3D model in these conditions.

All spheroid morphological parameters including area, roundness, circularity, sphericity and solidity extracted from 2D imaging were over 0.8 and stable over time (Figure S2).

Altogether, our ULA method maintains the viability of RL cells in 3D and spheroid morphological features over time.

### 3.3. Determination of Optimal Cell Density for Drug Testing

Spheroids are relevant and powerful models for testing drug sensitivity compared to cell suspension cultures [6,13,38,39,40]. In order to evaluate the most favorable cell seeding density for the drug assay, we first tested ABT-199 (a Bcl2 inhibitor) a classical apoptosis inducer in FL. Global morphology was observed by bright field imaging and cell death determined by propidium iodide labeling on entire ULA-MALC with an automated confocal microscope. After 3 days of treatment, ABT induced a concentration-dependent reduction of ULA-MALC size at the three different cell seeding densities tested, compared to the untreated condition (Figure 3A). These effects were more pronounced after 6 days of treatment (Figure S3). The basal level of cell death observed in the untreated condition was also increased confirming results obtained by flow cytometry (Figure 1C and Figure 3A). Several morphological parameters were extracted such as area, and cell death induction estimated by the area and intensity of incorporated propidium iodide. After 3 days of treatment at a 2500 cell seeding density, ABT-199 at 100 nM induced a significant reduction of ULA-MALC area compared to the untreated condition (Figure 3B). This was concomitant with an increase of cell death as attested by the increase of propidium iodide area and intensity (Figure 3C). Flow cytometry performed on dissociated ULA-MALC confirmed that ABT-199 induced cell death (7AAD^+^ cells) in a dose-dependent manner (Figure 3D). Experiments performed with 5000 or 10,000 cells exhibited similar results suggesting that cell density did not influence ABT-199 responses (Figure 3).

Altogether, these experiments showed that 2500 cells represent the most suitable cell seeding density for developing regular, spherical ULA-MALC exhibiting a very low basal level of cell death.

### 3.4. Drug Sensitivity Testing

We then determined whether the ULA-MALC model was adapted for a larger scale drug testing assay. Thus, we tested five different drugs known to directly target different pathways in FL [2,41] and determined their efficacy based on: i) their impact on morphology (area and roundness) measurable on entire ULA-MALC and ii) cell death induction quantified in a dissociated or whole 3D model. Each molecule was also tested in the presence of RTX or GA101 as used in the clinic [2,42]. Here we detailed the results obtained after 3 days of treatment (Figure 4). Observation of ULA-MALC by bright field microscopy (Figure 4A) and analysis of area (Figure 4B left panel) using an automated confocal microscope revealed that all drugs, except ibrutinib, strongly reduced ULA-MALC area compared to the untreated condition. RTX and GA101 alone had a potent effect on the area by reducing BF area by 62% and 73% respectively. However, a combination with drugs did not enhance their effect. Roundness was only affected when drugs were combined with GA101 (except for ibrutinib) (Figure 4B, right panel). Moreover, we were able to visualize (Figure 4A) and quantify (Figure 4C) propidium iodide incorporation in whole ULA-MALC treated or not with anti-CD20 mAbs combined with drugs. At the basal level, we observed propidium iodide labeling mainly in the periphery of the ULA-MALC. This pattern was similar in lenalidomide, ibrutinib and bendamustine treated conditions. However, in ULA-MALC treated with the rapamycin/anti-CD20 mAbs combination, doxorubicin and, as expected, in the positive control (ABT-199), propidium iodide distribution was more potent and diffuse. Anti-CD20 mAbs induced a diffuse propidium iodide distribution in ULA-MALC with a more potent labeling in the periphery in the presence or absence of drugs (Figure 4A). Propidium iodide intensity and propidium iodide area were determined in whole ULA-MALC. Except for GA101, none of the drugs tested induced an increase in cell death (Figure 4C). However, when cell death was measured by flow cytometry in dissociated ULA-MALC, we were able to detect an increase in 7AAD^+^ cells in doxorubicin and bendamustine treated ULA-MALC. Combination of RTX or GA101 with drugs did not modify the effects induced individually (Figure 4D). Although day 9 of culture exhibited a higher acceptable basal level of cell death (10% at 2500 cell seeding density) and result variability (Figure 1C,D), we also evaluated the effect of drugs after 6 days of treatment (Figure S4). A similar pattern was observed for bright field area with an effect observed for all drugs except ibrutinib. However, after 6 days of treatment, doxorubicin and bendamustin alone induced a significant decrease in ULA-MALC roundness that was not accompanied by propidium iodide intensity or area variation. Indeed, no variation in propidium iodide area was observed in all the conditions tested. In contrast, for some conditions such as RTX alone or in combination with rapamycin, and GA101 combined with rapamycin, ibrutinib or bendamustin, propidium iodide intensity significantly increased compared to the untreated condition (Figure S4). These discrepancies prompted us to develop 3D imaging and specific analysis to better characterize the 3D t(14;18)-positive B-NHL model and drug effects.

### 3.5. Three-Dimensional Imaging to Characterize the 3D Model

High-resolution images of intact spheroids, especially the inner layers, are very difficult to obtain by classical fluorescent microscopy, or confocal microscopy, due to the thickness of the model [43]. Classical optical microscopes allow a penetration depth of approximately 50–100 µm whilst the ULA-MALC diameter thickness varied between 325 and 1435 µm from day 1 to day 8 at 2500 cell seeding density suggesting an important thickness (Figure 2E). Thus, in depth 3D imaging using a light sheet fluorescent microscope (LSFM or SPIM for selective plane illumination microscope), which allows the highest penetration depth (>1 cm) compared to other optical sectioning microscopes [44], was necessary to achieve an in depth characterization of such a 3D model. Central image of SPIM z-stack showed a homogeneous cell distribution within ULA-MALC from day 1 to day 4, but at day 5 and day 6 we observed a difference of cell density between the peripheral layers of the ULA-MALC compared to the inner layers (Figure 5A, top panel). IMARIS tridimensional reconstruction of SPIM z-stack acquisitions from day 1 to day 6 allowed the visualization of the 3D structure of ULA-MALC and its evolution over time (Figure 5A, bottom panel). From day 1, the RL 3D model exhibited a flat side, a morphological feature impossible to observe by 2D imaging, suggesting that ULA-MALC were not perfectly spherical, in contrast to what we expected based on 2D imaging analysis (Figure 1C, SI > 0.9). This shape allowed us to define a maximum thickness/height starting from the flat bottom to the round top on a side view and a maximum diameter from a top view (Figure 5B). On day 1, thickness and diameter were close: 265 µm and 360 µm respectively (Figure 5B). Over time, ULA-MALC grew keeping this flat bottom, resembling a half sphere, and from day 4, the top of MALC began to flatten, with an exacerbation of the process at day 5 and day 6 (Figure 5A). These observations were confirmed by thickness and diameter quantifications (Figure 5B), with a thickness of MALC increasing up to day 3 (390 µm) and then decreasing over time (310 µm at day 6) whilst the diameter increased consistently, from 550 µm at day 3 to 1300 µm at day 6. These data suggested that this model reached a structural limitation in terms of thickness at 3 days of culture at this cell density, and increased only in terms of diameter after day 3. Although 2D imaging allowed the morphological characterization of ULA-MALC, 3D imaging and specific analyses appeared more adapted and necessary to study this model in more detail. Ki67 visualization at different layers from the top to the bottom (one image every 70 µm) confirmed the results obtained in Figure 2F where we observed a homogeneous distribution of proliferative cells in the whole ULA-MALC at day 3 of culture (Figure 5C and video S1).

The particular shape of MALC required dedicated quantification from 3D images to assess specific morphological properties. Thus, by developing dedicated algorithms, we were able to quantify the “real volume” and morphological properties of ULA-MALC over time. ULA-MALC real volume increased progressively from day 1 (0.028 mm^3^) to day 3 (0.069 mm^3^) and more rapidly at day 4 (0.14 mm^3^) and day 5 (0.30 mm^3^), doubling every 24 h (consistently with exponential cell proliferation, see Figure 2C), and slowing down at day 6 (0.35 mm^3^) (Figure 5D). Projected volume obtained with 2D imaging was dramatically different with an overestimation, especially from day 4 to day 6. Indeed, at day 6 of culture for example, real volume was 0.35 mm^3^, whilst the projected volume was estimated at 1.15 mm^3^ (Figure 5D).

The quantification of morphological properties confirmed that ULA-MALC were not perfect spheres as early as day 1, exhibiting a sphericity comprised between 0.72 and 0.78 and roundness between 0.75 and 0.88 from day 1 to day 4. After 5 days of culture, sphericity and roundness strongly decreased, with values dropping down to 0.25 and 0.23 respectively at day 6 (Figure 5E). With 2D images these values were close to 1 for both parameters (Figure 1B). We were also able to measure the eccentricity and showed an important ellipsoid deformation (very close to 1 at day 5 and day 6), in line with the drop in sphericity and roundness (Figure 5E). These results showed that both volume and sphericity dramatically changed between day 4 and day 5 with a huge increase in the real volume measured concomitantly to a potent loss in sphericity.

Altogether, the different results obtained from inverted and light sheet microscopes underlined the critical necessity to develop specific 3D imaging and dedicated analyses to realistically characterize the morphological properties of a 3D model.

### 3.6. Three-Dimensional Imaging to Evaluate Drug Sensitivity

Finally, we evaluated by SPIM the real direct effect of drugs after 3 days of treatment on whole ULA-MALC (Figure 6). Observation of the central image of SPIM z-stack (Figure 6A, top panel) showed that GA101 and ABT-199 treatment reduced the MALC diameter, although the peripheral cell layers were still more dense than the inner layers compared to the untreated condition. In contrast, we observed a potent decrease in volume after rapamycin treatment with a homogeneous effect on the ULA-MALC. This result, associated with the poor cell death induced by rapamycin (Figure 4), strongly suggests that this drug had a cytostatic effect on ULA-MALC, slowing the growth whereas the other drugs seemed to be cytotoxic. IMARIS tridimensional reconstructions of SPIM z-stack acquisitions confirmed that drug treatment altered the shape and volume of ULA-MALC (Figure 6A, bottom panel). Interestingly, GA101 seemed to compact the 3D structure as attested by the 3D reconstruction and central image from SPIM z-stack compared to the untreated condition, whilst the appearance of the rapamycin treated MALC was very close to the day 3/day 4 untreated ULA-MALC (Figure 5A). 3D image analysis showed that all the drugs did not significantly affect ULA-MALC thickness except for GA101 and ABT-199 100 nM for which we observed a slight increase (Figure 6B). In contrast, the diameter was significantly reduced after all treatments compared to untreated ULA-MALC with the highest effect observed in the presence of GA101 (Figure 6B). This was consistent with the impact on ULA-MALC growth observed by 2D imaging (Figure 4). Real volume quantification exhibited a significant and drastic drop induced by all treatments compared to untreated ULA-MALC (<0.15 mm^3^ vs. 0.35 mm^3^) (Figure 6C). Once again, the projected volume calculation led to a global overestimation in untreated and treated conditions, although the tendency was the same as for the real volume (drug treatments decrease MALC volume). However, more importantly, compared to real volume, it highlighted greater differences between untreated/treated conditions and between the different treated conditions (Figure 6C). Thus, volume quantification based only on diameter could potentially lead to important misinterpretations of drug effects in such 3D models.

In contrast, in 2D imaging, we showed that GA101, ABT-199 100 nM and rapamycin significantly increased sphericity (GA101, ABT-199 100 nM, rapamycin) and roundness (GA101 and rapamycin) (Figure 6D). The magnitude of this increase was consistent with the effect on ULA-MALC volume with the highest effect observed in GA101-treated MALC (Figure 6C). The effect of these drugs on these parameters was inversely correlated with their effect on eccentricity in agreement with our observation on thickness (Figure 6B). With regard to GA101, the effect was probably due to its potent cell aggregation property [45].

In conclusion, 3D characterization of treated ULA-MALC revealed important information as to drug effects on structuration, shape, volume and morphology. Although 2D imaging only allowed the observation of cytostatic or cytotoxic effects, 3D imaging highlighted other drug specific characteristics.

## 4. Discussion

Three-dimensional cultures are essential for cancer research as they allow cellular responses that more closely mimic those occurring in patients compared to cell suspension cultures. As their characteristics are closer to the native tumor microenvironment [3,46], these models represent powerful tools for studying pathology and drug efficacy. Techniques developed for 3D structure formation are numerous and depend on the type of experiment performed, but can be divided in two main types: scaffold-based and scaffold-free techniques. Both methods exhibit advantages and disadvantages explained in a very recent review [8]. These techniques are generally well documented in solid cancer cells with many articles dedicated to this field of research. However, this is not the case for lymphoma research, which benefits from a reduced number of publications (less than 150 referenced in PubMed). We are pioneers in the development of 3D models using t(14;18)-positive B-NHL cell lines and the HD technique to develop MALC (HD-MALC). Although this gave us a better understanding of the influence of spatial organization on gene and protein expression, drug distribution and efficacy in the presence or absence of immune cells [14,15,16,17,18], we cannot exclude that the addition of MC to favor cell aggregation does not influence cell signaling or behavior. Indeed, this may directly lead to alterations in growth and drug responses. To our knowledge, no study to date, has investigated the direct influence of MC, but, based on renal cell carcinoma cell line studies, it is known that both cell–cell interactions and O_2_ diffusion may be affected [47]. Another important point to consider is the use of 24-well plates in the HD method, which is not adapted for drug testing assays. Indeed, the transfer from the drop into agarose-coated wells was very difficult to perform. Considering both arguments, we adapted a scaffold free technique to our model using the ULA method and a centrifugation step allowing 100% of MALC formation. With this technique, cells do not display differences in gene and mutation expression profiles compared to the HD technique and 2D culture. Spheroids are not stressed by transfer, avoiding the risk of damaging the 3D structure. Moreover, ULA-MALC is centrally located in a non-agarose precoated well, which facilitates imaging. Altogether, this method seems to be reliable, robust, simple, it can be standardized and easily used for medium/high throughput screening for lymphoma therapies as described in solid cancers by others [6,10]. However, this is important to note that this model established with t(14;18)-positive B-NHL does not account for the contribution of the microenvironment and may be more representative of aggressive/transformed NHL, which can proliferate independent of surrounding cells. Nevertheless, it can be complexified by adding immune cell such as gamma delta T lymphocytes [17], NK cells [18] or cells from the tumor microenvironment such as macrophages or dendritic cells.

We determined the most appropriate seeding cell density for ULA-MALC formation based on morphological parameters visualized by phase contrast microscopy and extracted with a macro adapted from Ivanov and colleagues [13]. ULA-MALC grew fast, and appeared round, spherical and regular when their indexes were higher than 0.9 according to Zanoni [13] and Santo [48], for all densities tested. We observed a slight drop in spheroid roundness after 6 days of culture when 10,000 cells were used to elaborate the ULA-MALC. As described by Gong and colleagues [49], this may be due to an increase in cell death as observed on entire ULA-MALC after PI staining or dissociated ULA-MALC after 7AAD labeling. Cell death is a physiological phenomenon in spheroids due to the absence of vasculature, the presence of a hypoxic core and cells at different stages of maturation in different locations (viable, proliferative and quiescent) [12,35]. The drop of roundness was also correlated with an increase in the total spheroid area and an increase of SD attesting a higher variability between the experiments when this cell density was used. Thus, the 2500 cell seeding density and day 3 of culture appear to be the most adaptable for drug treatment as viable cells are homogeneously distributed and this avoids the loss of sphericity and compactness.

ULA-MALC enters rapidly in a spheroidization process exhibiting a round-type morphology probably due to strong cell–cell interactions [13]. Moreover, ECM, which is increased in 3D compared to 2D t(14;18)-positive B-NHL cell culture [15], may ensure total compactness. This was not the focus of our study, but it would be interesting to investigate the kinetics of ECM production in ULA-MALC and to determine its role in the spheroidization process. Three-dimensional culture may influence the response to treatment. In solid cancers, it is well established that 3D cultures are more resistant than cell suspensions because of the lower drug accessibility, the activation of genes involved in survival and drug resistance in response to hypoxia and the low rate of proliferating cells (for drugs that are active against proliferating cells) [8]. In t(14;18)-positive B-NHL, we observed that the response to anti-CD20 mAbs was different between 3D cultures (HD-MALC) and 2D cultures [15]. In this study, we investigated whether the density could impact direct drug responses using a classical inhibitor of FL cells. ABT-199 was efficient in decreasing area and cell death in ULA-MALC whatever the seeding cell density used. This confirms that cell density and/or spheroid diameter does not influence the drug response. The mechanisms that could explain the discrepancies between solid and hematological cancers are not known. For small pharmacological inhibitors, the drug can homogeneously penetrate spheroids whatever its volume and induce a similar effect. However, unpublished data from our group suggests that MALC volume strongly influences anti-CD20 mAbs efficacy reflecting pre- and clinical observations [50,51,52].

Another critical point highlighted in our study is the level of cell death measured by flow cytometry, which did not correlate with the level observed and measured by 2D imaging. This may be due to the fact that for flow cytometry, ULA-MALC are dissociated and that the percentage of cell death is measured as the mean fluorescent intensity (i.e., 7AAD) per 50,000 cells. In contrast, for 2D imaging, the operetta system allows light to penetrate at a depth ranging from 50 to 100 µm. The fluorescent intensity (propidium iodide) measured therefore represents the accumulation of signal that the microscope can get from the first layers of the ULA-MALC. Moreover, although we can acquire several images at different levels on the z-axis (z-stack) and achieve a maximum projection with the associated software (Columbus), we can only obtain partial information corresponding to the top of the sample, leading to misinterpretation. The Operetta system is considered to be a high-content imaging system, providing high-resolution, high sensitivity and high speed required for 3D cell-culture model imaging. With our study, we identified two major limitations for ULA-MALC imaging using this equipment. Firstly, our system is not equipped with a water immersion objective and secondly, ULA-MALC are not cleared, which strongly limits the penetration. Clearing methods are numerous, aqueous or solvent-based, and necessary for in depth imaging of large samples such as spheroids [53,54]. They have been classically developed for light sheet microscopy but also for confocal and multiphoton microscopy [31,55,56]. We tried aqueous methods to attempt to clear MALC in the ULA 96 well plates but unfortunately, we did not yet obtain satisfactory results. We are still working to develop a new process allowing a global workflow in 96 well plates comprising clearing steps that avoid manipulation or transfer into new devices.

In parallel, SPIM, which exposes a sample to up to 5000 times less energy than confocal fluorescence microscopy, minimizes photo-bleaching and photo-induced cell damage. Light sheet illumination is becoming an important tool for spheroid, organoid, tissue and small animal imaging. It holds great promises for the analysis of large numbers of samples with simple preparation, fast recording speed, high resolution and multi-channel fluorescence imaging [57]. High throughput screening/imaging with SPIM is under development [58,59]. This technical improvement has triggered great interest on behalf of the “spheroid community” and accessible and commercial devices are up and coming. This will bridge the gap between high content screening 2D imaging and precise in-depth 3D imaging.

With the SPIM and specifically developed algorithms, we were able to determine the real morphology of ULA-MALC, which is, in contrast to the information obtained from the automated confocal microscope, non-spherical. According to Zanoni [13], untreated ULA-MALC are not “spherical” but are more “ellipsoidal” between day 1 and day 4 and become “irregular” after 5 days of culture. This is confirmed by the 3D reconstruction observed after SPIM imaging and the increase in the eccentricity index, which mirrors the decrease in sphericity. The drop in roundness observed by SPIM during culture time corresponds to an increase in cell death and a possible lesser compaction leading to an increase in total area. We also show here that SPIM imaging is crucial to morphologically characterize the effect of drugs. Now, it should be interesting to investigate the localization of drug-induced cell death in a working window where ULA-MALC are more spherical and compact (day 2-day 4) and where images can be acquired by classical SPIM.

Here we developed a new technique to generate t(14;18)-positive B-NHL 3D cell culture that is easy to handle, inexpensive, robust and reliable. This method allows, by a simple workflow, an increase in the throughput for drug testing offering new perspectives for preclinical studies. Moreover, our study reveals potent discrepancies between the results obtained from 2D and 3D imaging and warns the scientific community as to possible misinterpretations following 2D imaging. Numerous investigations using spheroids are based on 2D imaging performed on uncleared samples without any complementary in depth characterization of the model used, nor the drug effect induced. We show here that without 3D imaging and development of specific informatics solutions, we can draw wrong conclusions.

## 5. Conclusions

Altogether, this study proposed a new method, imaging tools and informatic solutions developed for t(14;18)-positive B-NHL, but also warned the general spheroid community. By improving such investigations on 3D models, we contributed to the development of alternative assays to reduce animal testing and costs, which is necessary for future preclinical studies. This study opened encouraging perspectives in terms of model development for the use of coculture with immune cells to explore the activity of immunotherapy including external effectors. Moreover, this work established the methodological basis and workflow for the development of patient derived spheroids (PDLS) integrating tumor microenvironment. This may allow in the future a better B-NHL biological characterization and preclinical studies in a more relevant and complexified system better recapitulating disease heterogeneity and microenvironment contribution.

## Data Availability

The supplementary data are available here.

## Supplementary Materials

The following are available online at https://www.mdpi.com/2072-6694/13/7/1490/s1, Figure S1: Influence of cell density on ULA-MALC biology for SC1, DOHH2 and WSU-NHL cell lines, Figure S2: Morphological 2D characterization of ULA-MALC at 2500 cell seeding density, Figure S3: Influence of cell density on drug response after 6 days of treatment, Figure S4: Drug response of ULA-MALC after 6 days of treatment, Table S1: Statistics for Figure 3, Figure 4 and Figure 6, Video S1: Visualization of Ki67 labeling by SPIM.
