# Supplementary material for: 3D Model Characterization by 2D and 3D Imaging in t(14;18)-Positive B-NHL: Perspectives for In Vitro Drug Screens in Follicular Lymphoma
Source: Cancers (Basel). 2021 Mar 24;13(7):1490. doi: 10.3390/cancers13071490 (PMC8036410; doi:10.3390/cancers13071490)
Supplement: Supplementary file 1 [file cancers-13-01490-s001.zip › cancers-1110957 supplemenraty after proof.docx]

Supplementary Materials: 3D Model Characterization by 2D and 3D Imaging in t(14;18)-Positive B-NHL: Perspectives for in vitro Drug Screens in Follicular Lymphoma

Fabien Gava, Carla Faria, Pauline Gravelle, Juan G Valero, Cèlia Dobaño-López, Renaud Morin, Marine Norlund, Aurélie Gomes, Jean-Michel Lagarde, Cédric Rossi, Julie Bordenave, Laetitia Pieruccioni, Jacques Rouquette, Alba Matas-Céspedes, Jean-Jacques Fournié, Loïc Ysebaert, Camille Laurent, Patricia Pérez-Galán and Christine Bezombes

**B**

**Figure S1.** Influence of cell density on ULA-MALC biology for SC1, DOHH2 and WSU-NHL cell lines. ULA-MALC from SC1, DOHH2 and WSU-NHL cell lines were cultured at different cell seeding densities (2500; 5000; 10,000 cells) and different biological characteristics were determined at different culture times. (**A**) Growth and morphology observed by bright field (BF) microscopy (Cytation™) at 4× after 3, 6 and 8 days (D) of culture. Scale: 1200 µm. These pictures are representative of 3 independent experiments comprising 12–20 individual ULA-MALC (depending on the cell line and the condition). (**B**) Morphological properties (projected area, sphericity) determined after 1, 3, 6 and 8 days (D) of culture by 2D imaging analysis with the specific macro developed (see Methods section). These graphs are the mean ± SD of *n* =3 independent experiments comprising *n* = 12–20 individual ULA-MALC (depending on the cell line and the condition). (**C**). Cell death quantification on whole ULA-MALC at day 6 and 9 after propidium iodide (PI) labeling and 2D imaging (as Figure 2C, also see Methods section). Experiment performed on 3 independent experiments comprising 8–20 individual ULA-MALC (depending on the cell line and the condition). Mean ± SD of the ratio of propidium iodide positive area (PI) in relation to the total bright field (BF) area. (**D**) Cell death measured by flow cytometry (Attune focusing acoustic cytometer and software, Life Technologies) after 7AAD staining of dissociated MALC. This graph represents mean ± SD of the percentage of cell death (7AAD+) measured in 2 independent experiments of 3 pooled ULA-MALC.

**Figure S2.** Morphological 2D characterization of ULA-MALC at 2500 cell seeding density. Area, circularity, roundness, solidity and sphericity of ULA-MALC established with RL cells measured at the indicated times of culture by 2D imaging (D = day). These graphs represent the mean ± SD of 3 independent experiments each comprising at least 30 individual ULA-MALC.

**Figure S3.** Influence of cell density on drug response after 6 days of treatment. ULA-MALC established with RL cells were seeded at different cell densities (2500; 5000 and 10,000 cells) and treated or not (UT) at day 3 of culture with ABT-199 (ABT) at 10 nM and 100 nM. Cell death was visualized and measured 6 days post-treatment (ie D9 of culture). Results are representative (pictures) or the mean ± SD (graphs) of 3 independent experiments each comprising 5 individual ULA-MALC. *p* values: **** = *p* <0.0001, *** = *p* <0.0005, ** = *p* <0.01, * = *p* <0.05. (**A**). Global morphology (BF) and propidium iodide (PI) labeling were visualized by 2D imaging. **(B).** Bright field (BF) area measured. Results represent the mean ± SD of the global BF area normalized to the untreated condition (UT). (**C**). Quantification of cell death after PI labeling on whole ULA-MALC cultured at 2500, 5000, 10,000 cell seeding densities and treated or not (UT) with ABT-199. Results represent the mean ± SD of the global BF area (upper graphs), PI area in relation to BF area (middle graphs) and PI intensity in relation to BF area (lower graphs), all normalized to the untreated condition (UT). (**D**). Cell death measured by flow cytometry in dissociated ULA-MALC cultured at 2500; 5000 and 10,000 cell seeding densities and treated or not (UT) with ABT-199. Results represent the mean ± SD of the percentage of cell death (7AAD^+^).

**Figure S4.** Drug response of ULA-MALC after 6 days of treatment. ULA-MALC established with RL cells were seeded at 2500 cells and treated or not (UT) after 3 days of culture with rapamycin (Rapa, 10 nM), doxorubicin (Doxo, 0.1 µM), lenalidomide (Len, 5 µM), ibrutinib (Ibru, 500 nM), bendamustin (Benda, 10 µg/mL) in combination or not with RTX or GA101 (10 µg/mL). ABT-199 (ABT) at 100 nM was used as positive control. Figures represent results obtained after 6 days of treatment and are representative (pictures) or the mean ± SD (graphs) of 3 independent experiments each comprising 5 individual ULA-MALC. *p* values: **** = *p* <0.0001, *** = *p* <0.0005, ** = *p* <0.01, * = *p* <0.05. (**A**). Visualization of global morphology (BF) and propidium iodide (PI) labeling by 2D imaging. (**B**). Bright field (BF) area and roundness determined by 2D imaging analysis with the specific macro developed (see Methods section). Results represent the mean ± SD. (**C**). Cell death quantification after PI labeling by 2D imaging on whole ULA-MALC. Results represent the mean ± SD of PI area in relation to BF area (left) and PI intensity in relation to BF area (right), all normalized to the untreated condition (UT). (**D**). Cell death quantification by flow cytometry in dissociated ULA-MALC. Results represent the mean ± SD of the percentage of cell death (7AAD^+^).

**Table S1. Statistics for Figure 3, Figure 4 and Figure 6.**

Statistical testing is represented as tables (rows against columns). Normality tests were performed before Tukey’s multiple comparisons tests (one-way ANOVA). p values: **** = *p* < 0.0001, *** = *p* < 0.0005, ** = *p* < 0.01, * = *p* < 0.05

Figure 3: 2500 cells

- D6D3pttt: figure 3 B–D

|  | **Area IP/area BF** | | **Intensity IP/area IP** | | **7AAD** | | **Area BF** | |
| --- | --- | --- | --- | --- | --- | --- | --- | --- |
|  | ABT10 nM | ABT100 nM | ABT10 nM | ABT100 nM | ABT10 nM | ABT100 nM | ABT10 nM | ABT100 nM |
| UT | **** | **** | ns | ** | ns | *** | ns | *** |

- D9D6pttt: supplemental figure 4 B–D

|  | **Area IP/area BF** | | **Intensity IP/area IP** | | **7AAD** | | **Area BF** | |
| --- | --- | --- | --- | --- | --- | --- | --- | --- |
|  | ABT10 nM | ABT100 nM | ABT10 nM | ABT100 nM | ABT10 nM | ABT100 nM | ABT10 nM | ABT100 nM |
| UT | ** | *** | * | ** | * | ** | **** | **** |

Figure 3: 5000 cells

- D6D3pttt: figure 3 B–D

|  | **Area IP/area BF** | | **Intensity IP/area IP** | | **7AAD** | | **Area BF** | |
| --- | --- | --- | --- | --- | --- | --- | --- | --- |
|  | ABT10 nM | ABT100 nM | ABT10 nM | ABT100 nM | ABT10 nM | ABT100 nM | ABT10 nM | ABT100 nM |
| UT | **** | **** | **** | **** | ** | *** | *** | **** |

- D9D6pttt: supplemental figure 4 B, C, D

|  | **Area IP/area BF** | | **Intensity IP/area IP** | | **7AAD** | | **Area BF** | |
| --- | --- | --- | --- | --- | --- | --- | --- | --- |
|  | ABT10 nM | ABT100 nM | ABT10 nM | ABT100 nM | ABT10 nM | ABT100 nM | ABT10 nM | ABT100 nM |
| UT | *** | **** | ns | *** | ns | * | **** | **** |

Figure 3: 10,000 cells

- D6D3pttt: figure 3 B–D

|  | **Area IP/area BF** | | **Intensity IP/area IP** | | **7AAD** | | **Area BF** | |
| --- | --- | --- | --- | --- | --- | --- | --- | --- |
|  | ABT10 nM | ABT100 nM | ABT10 nM | ABT100 nM | ABT10 nM | ABT100 nM | ABT10 nM | ABT100 nM |
| UT | **** | **** | *** | *** | ns | ** | **** | **** |

- D9D6pttt: figure S4 B–D

|  | Area IP/area BF | | Intensity IP/area IP | | 7AAD | | Area BF | |
| --- | --- | --- | --- | --- | --- | --- | --- | --- |
|  | ABT10 nM | ABT100 nM | ABT10 nM | ABT100 nM | ABT10 nM | ABT100 nM | ABT10 nM | ABT100 nM |
| UT | *** | **** | * | **** | * | ** | **** | **** |

Figure 4: 2500 cells

- D6-D9pttt: Figure 4 B–D

| **UT VS** | **Area BF** | **Roundness** | **Area IP/area BF** | **Intensity IP/area BF** | **7AAD** |
| --- | --- | --- | --- | --- | --- |
| RTX | **** | ns | ns | ns | ns |
| GA | **** | ns | **** | **** | ns |
| Rapa | **** | ns | ns | ns | ns |
| R+R | **** | ns | ns | ns | ns |
| R+G | **** | **** | **** | **** | ns |
| Doxo | **** | ns | ns | ns | **** |
| Doxo + R | **** | ns | **** | ns | **** |
| Doxo + G | **** | *** | **** | *** | **** |
| Ibru | ns | ns | ns | ns | ns |
| Ibru + R | **** | ns | ns | ns | ns |
| Ibru + G | **** | ns | **** | **** | ns |
| Lena | **** | ns | ns | ns | ns |
| Lena + R | **** | ns | **** | * | ns |
| Lena + G | **** | ** | **** | * | ns |
| Benda | **** | ns | ns | ns | *** |
| Benda + R | **** | ns | ns | ns | **** |
| Benda + GA | **** | **** | **** | **** | **** |
| ABT | **** | **** | **** | **** | **** |

- D9-D6pttt : supplemental figure 4 B, C, D

| **UT VS** | **Area BF** | **Roundness** | **Area IP/area BF** | **Intensity IP/area BF** | **7AAD** |
| --- | --- | --- | --- | --- | --- |
| RTX | **** | ns | ns | * | ns |
| GA | **** | ns | ns | ns | ns |
| Rapa | **** | ns | ns | ns | ns |
| R+R | **** | ns | ns | **** | ns |
| R+G | **** | ns | ns | **** | ns |
| Doxo | **** | **** | ns | ns | **** |
| Doxo + R | **** | **** | ns | ns | **** |
| Doxo + G | **** | **** | ns | ns | **** |
| Ibru | ns | ns | ns | ns | ns |
| Ibru + R | **** | ns | ns | ns | **** |
| Ibru + G | **** | ns | ns | ** | ns |
| Lena | **** | ns | ns | ns | ns |
| Lena + R | **** | ns | ns | ns | ns |
| Lena + G | **** | * | ns | ns | ns |
| Benda | **** | * | ns | ns | **** |
| Benda + R | **** | *** | ns | ns | **** |
| Benda + GA | **** | **** | ns | ** | **** |
| ABT | **** | ns | **** | **** | **** |

Figure 6 Tests:

- Normality tests not passed, non-parametric tests

- Side-by-side Mann-Whitney tests, untreated (UT) versus each drug

- *p* values: **** = *p* < 0.0001, *** = *p* < 0.0005, ** = *p* < 0.01, * = *p* < 0.05

- ns : non-significant (*p* > 0.05)

- *n* were too small for Bendamustin and GA101+Bendamustin to perform statistical test (*n* = 2)

Figure 6B

- Maximum Thickness:

|  | **ABT10 nM** | **ABT100 nM** | **GA101** | **Rapa** |
| --- | --- | --- | --- | --- |
| UT | ns | *** | **** | ns |

- Maximum Diameter:

|  | **ABT10 nM** | **ABT100 nM** | **GA101** | **Rapa** |
| --- | --- | --- | --- | --- |
| UT | ** | **** | **** | **** |

Figure 6C

- Real Volume:

|  | **ABT10 nM** | **ABT100 nM** | **GA101** | **Rapa** |
| --- | --- | --- | --- | --- |
| UT | ** | *** | **** | *** |

- Projected Volume:

|  | **ABT10 nM** | **ABT100 nM** | **GA101** | **Rapa** |
| --- | --- | --- | --- | --- |
| UT | ** | **** | **** | **** |

Figure 6D

- Sphericity:

|  | **ABT10 nM** | **ABT100 nM** | **GA101** | **Rapa** |
| --- | --- | --- | --- | --- |
| UT | ns | * | **** | *** |

- Roundness:

|  | **ABT10 nM** | **ABT100 nM** | **GA101** | **Rapa** |
| --- | --- | --- | --- | --- |
| UT | ns | ns | *** | ** |

- Mean Eccentricity:

|  | **ABT10 nM** | **ABT100 nM** | **GA101** | **Rapa** |
| --- | --- | --- | --- | --- |
| UT | ns | ** | **** | *** |

Supplementary Video S1 - Visualization of Ki67 labeling by SPIM

Ki67 visualization in whole ULA-MALC (2500 cell seeding density, day 3 of culture) by SPIM at 5X magnification, scale: 200 µm, 7 frames per second (FPS).
